# Plant-Origin Stabilizer as an Alternative of Natural Additive to Polymers Used in Packaging Materials

**DOI:** 10.3390/ijms22084012

**Published:** 2021-04-13

**Authors:** Angelika Plota, Anna Masek

**Affiliations:** Institute of Polymer and Dye Technology, Faculty of Chemistry, Lodz University of Technology, Stefanowskiego 12/16, 90-924 Lodz, Poland; angelika.plota@gmail.com

**Keywords:** cannabidiol (CBD), natural additives, weathering, polylactide, ethylene-norbornene copolymer, packaging materials

## Abstract

Over the past 25 years, cannabis plants have gained major popularity in the research community. This study aimed to evaluate the antioxidant capacity and stabilization efficiency of cannabidiol (CBD) extract in two different polymers: polylactide (PLA) and ethylene–norbornene copolymer (Topas) that are used in packaging materials more often. The research technology included weathering in a special chamber, surface free energy and color change measurements, surface morphology and Fourier-transform infrared spectroscopy (FTIR) analysis, thermogravimetry, and determination of the oxidation induction time or temperature (OIT) values, based on which the effectiveness of the cannabidiol extract could be estimated. Obtained results showed that the addition of CBD to polymer mixtures significantly increased their resistance to oxidation, and it can be used as a natural stabilizer for polymeric products. Moreover, samples with cannabidiol changed their coloration as a result of weathering. Therefore, this natural additive can also be considered as a colorimetric indicator of aging that informs about the changes in polymeric materials during their lifetime. On the other hand, surface properties of samples with cannabidiol content did not alter much compared to pure Topas and PLA.

## 1. Introduction

Due to the light weight, low cost, and high efficiency of plastics, combined with easy processability, their deployment in packaging materials has grown significantly in recent years [1]. Moreover, packaging must meet requirements, such as non-toxicity, water vapor and oxygen impermeability, transparency, and good mechanical properties. Therefore, polymers have prevailed in the wrappings market. By 2050, the production of plastic packaging is anticipated to exceed 250 million metric tons [2,3]. This forecast has become disturbing due to serious environmental pollution problems. Nowadays, millions of tons of plastic wastes (including plastic bottles, plastic bags, or food wrappers) accumulate in landfills and pollute coastlines and oceans [4,5]. The reason is the short service life of these products, which in some cases is less than a week, and their great durability. Most of the synthetic polymeric materials are designed for good strength and efficiency without taking into account their degradability or recyclability. Therefore, scientists have attempted to produce new materials that are more environmentally friendly or whose degradation process can be controlled [6,7,8,9,10].

An important strategy that has been developed over the past twenty years focuses on the possibility of using biodegradable plastics in packaging materials, which definition was standardized by ISO/TC61/SC5/WG22 (ISO 472/DAM3, Amendment 3, General Terms and Terms Relating to Degradable Plastics) [6]. The feature of these products is their complete degradation by water and carbon dioxide as a result of naturally occurring microorganism activity (e.g., fungi, bacteria, algae) [2,6,11,12,13]. The most common and the most widely used among all biobased and biodegradable polymeric materials investigated to date are polylactide (PLA) and polyhydroxyalkanoates (PHAs). Both belong to the aliphatic polyesters group, and what is important, they are characterized by good mechanical properties that are similar to those for synthetic polymers, such as polystyrene (PS), polyethylene (PE), or polypropylene (PP) [8,14,15]. Moreover, they can be obtained from renewable resources, e.g., from sugars or starch, and the most important advantage that differentiate PLA and PHAs from traditional plastics is their total biodegradability under a wide range of conditions in the natural environment [3,8,16,17,18,19]. Due to their ecological character and satisfactory durability, these polymers are mainly used in packaging and disposable products. However, their technology is constantly developed with the intention of using them as materials for agricultural engineering, such as sandbags, as materials for fisheries, e.g., as fishing nets, and in the medical context as bioabsorbable products [6].

On the other hand, not only the type of polymer matrix but also the additives play a key role in human health and the environment. Polymeric materials are subjected to changes in their properties during the exploitation period because of various degradative factors, such as higher temperature, UV radiation, as well as humidity. Therefore, specific stabilizers which protect the polymer matrix and thus slow down its degradation process are used. The latest scientific reports show the benefits of using natural origin and non-toxic additives to polymers. Previously, synthetic antioxidants, such as butylated hydroxyanisole (BHA) or butylated hydroxytoluene (BHT), were widely applied in food packaging materials to prevent lipid oxidation, but now, they are increasingly replaced by compounds such as polyphenols, plant extracts, or essential oils [20,21,22]. Their big advantage is not only that they increase the ecological profile of the product, thanks to which it meets the requirements that are more and more restrictive in the packaging industry, but also as a result of their ability to work as radical scavengers, they protect the polymer during its service life, preventing its oxidation process [23,24,25,26,27,28].

However, in the case of single-use products, their long life is not a desirable attribute. Taking into account the environment, a good alternative to give biodegradability to products made of synthetic polymers, e.g., polyolefins, is the addition of the prooxidant compounds into the polymeric matrix. Additives with prooxidative properties include oxidation initiators that originate the oxidation of the material when it is subjected to heat or light. As a result of this process that concurs with the destruction of the macromolecules in the polymer, the entire product is characterized by a lower molecular weight. An example of these compounds is stearates of transition metals. Furthermore, in some cases, an antioxidant can also act as a prooxidant. For instance, α-Tocopherol (vitamin E) is known as a powerful antioxidant, but depending on the concentration used, it may also exhibit prooxidative properties as a result of its antioxidant mechanism. When this compound reacts with free radicals, and there is not enough ascorbic acid, which is needed for its regeneration, then it becomes a radical itself, and the autooxidation of linoleic acid is promoted [29,30,31,32,33].

In the following study, two different polymers, polylactide (PLA) and ethylene–norbornene copolymer (Topas), were used as polymer matrices. Polylactide is a bio-based polymer marked by good biodegradability, biocompatibility, and sustainability, which can also be a green alternative for synthetic polymers. However, Topas, which belongs to cyclic olefin copolymers (COCs), is characterized by low permeability to gases and moisture, high transparency, and very high purity [34,35]. Therefore, both are more often used in packaging materials. Moreover, over the past 25 years, cannabis plants have gained major popularity in the research community because of the discovery of their huge potential not only in medicine but also in skincare cosmetics. These plants contain a system responsible for the production of molecules labeled as cannabinoids, flavonoids, and terpenes. Cannabidiol (CBD) is a non-psychotropic component of the plant that exhibits anti-inflammatory and, above all, antioxidant properties due to the presence of two hydroxyl groups. For this reason, in this work, an attempt to investigate the antioxidant effect of this compound on the oxidation process of PLA and ethylene-norbornene copolymer (Topas) was performed [36,37,38]. Moreover, in the available literature, CBD extract has not been described as a potential natural additive to polymers so far. Therefore, this research may not only contribute to expanding the current state of knowledge but also present the possibility of using natural additives that are not harmful to both the environment and human health.

## 2. Results and Discussion

### 2.1. Physicochemical Properties of Ethylene–norbornene Copolymer (Topas) and Polylactide (PLA) Composites with Cannabidiol (CBD) Extract

**Figure 1:**
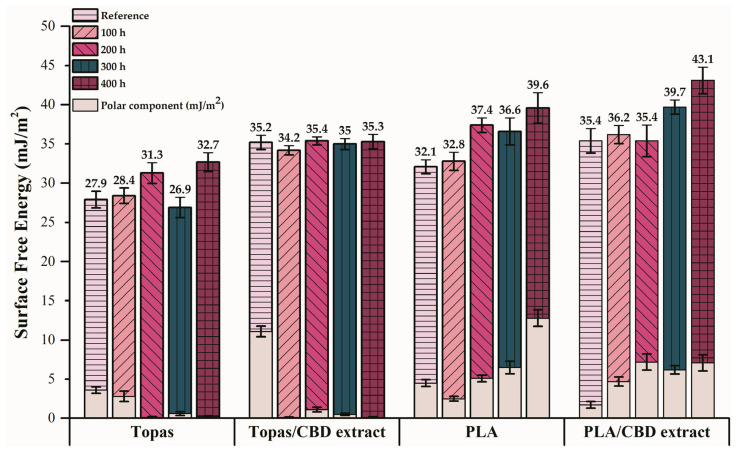
Changes caused by weather-aging in surface free energy (SFE) of pure ethylene–norbornene copolymer (Topas), polylactide (PLA), and these polymers with cannabidiol (CBD) content.

The first part of the study was to examine the surface free energy (SFE) of all samples that present the surface tension of a solid and are formed as a result of molecular interactions occurring between the air and the solid phase. In many applications, it is a crucial parameter that indicates how the solid behaves in contact with fluids, e.g., an assessment of surface coverage with paints or varnishes. Furthermore, sometimes it can also be considered as an indicator of the polymer’s susceptibility to oxidation processes. In general, when the SFE of the solid is high, it means a good wettability by any liquid. In this study, the Owens–Wendt–Rabel–Kealble theory was used, which consists of the contact angles determination by applying at least two liquids with different polarities and divides the surface energy into two constituents: polar and dispersive, respectively. The surface free energy was determined for all samples before and after 100, 200, 300, 400 h of controlled weather-aging based on distilled water, diiodomethane, and ethylene glycol contact angles values. For both polymers, the effect of the 1 phr (parts per hundred rubber) cannabidiol extract addition to tested composites on their surface properties was investigated. For each material, 10 contact angles of the three liquids with different polarities were marked. Obtained results are presented in Figure 1 and Table 1. Some of the main non-toxic phytocannabinoids are cannabidiol (CBD) and cannabidiolic acid [39]. Phytochemical compounds are sensitive to UV radiation that can result in their polymerization or degradation process, which can be a reason for changes in their surface properties.

Analyzing the calculated surface energy for pure Topas and Topas with CBD samples, an increase in the surface free energy (SFE) values can be observed with a longer aging time. For Topas-CBD blends, the difference in the obtained surface energy results before and after 400 h of aging was really slight (0.1 mJ/m^2^), which means that they are characterized by good resistance to oxidation. However, the aging effect on the surface properties of pure Topas samples was more visible. The difference in the SFE value between the reference specimen and after 400 h of weathering was equal to 4.8 mJ/m^2^. Therefore, the cannabidiol additive turned out to be effective by slowing down the degradation of Topas samples. Moreover, taking into account the polar components of the surface free energy, both for the Topas and Topas-CBD blends, there was a significant decrease in these values, which proves their considerable resistance to the oxidizing agent. It can be assumed that this polymer cross-linked during the weather-aging, which resulted in a significant reduction in the surface energy polar component. Additionally, analyzing the obtained contact angles in the case of distilled water as a measurement liquid (Table 1), an increase in these values for Topas-based samples was observed. In general, when a contact angle for water is less than 90°, it signifies that the material has a hydrophilic nature and is more susceptible to oxidation, and when it is above 90°, it means more hydrophobic character and better resistance to the oxidation process [40]. Therefore, the weathering effect on the surface properties of the pure ethylene–norbornene copolymer and with CBD content turned out to be nonsignificant, and products made of this polymer can be characterized by long-term use.

On the other hand, for pure PLA and PLA with CBD mixtures, the changes in surface properties due to aging were much higher, and in both cases, comparing the samples before and after weathering, a major increase in the SFE values was observed. Furthermore, analyzing the polar constituents of the surface energy, for pure polylactide, this value was equal to 12.8 mJ/m^2^ after 400 h of aging, which was almost three times larger in comparison to the reference sample (4.5 mJ/m^2^). However, in the case of PLA with CBD content, the difference in polar components of the SFE before and after 400 h of weathering was much lower, which can indicate that the cannabidiol extract turned out to be effective for preserving its surface properties. Additionally, for all PLA samples, the obtained contact angle values for distilled water as a measurement liquid were much lower than 90°, which means that polylactide is characterized by a hydrophilic nature and higher susceptibility to oxidation processes. These were improved a little as a result of the CBD extract addition.

However, apart from distilled water, diiodomethane and ethylene glycol were also used as measuring liquids. The obtained values for these fluids were much lower than in the case of distilled water. This was due to the surface tension and the polar and dispersive components of water, diiodomethane, and ethylene glycol, which are known. In general, water is a highly polar liquid, the polar component of which is 51.0 mJ/m^2^, and the total SFE value is 72.8 mJ/m^2^. For diiodomethane, the SFE constituents were as follows: polar, 2.4 mJ/m^2^; dispersive, 48.6 mJ/m^2^, whereas for ethylene glycol, the polar and dispersive components were equal to 16.8 mJ/m^2^ and 30.9 mJ/m^2^, respectively [41]. As it can be observed, in most cases, for Topas-based composites, the contact angle values for diiodomethane and ethylene glycol were higher than for PLA samples with and without CBD extract. It can be supposed that it is also related to the better aging resistance of Topas. But it should be highlighted that most scientific papers are focused only on the water contact angles’ behavior because they give an indication of the wettability of the solid and inform about the susceptibility to oxidation. The usage of diiodomethane and ethylene glycol was mainly necessary for a regression line while determining the surface free energy. Gonçalves et al. (2013) [42] investigated the effect of natural and synthetic antioxidants (α-tocopherol, butylated hydroxytoluene, and tert-butylhydroquinone) used in different amounts on surface properties of polylactide (PLA). To assess the wettability parameter, they used water as a measuring liquid, and the surface energy of the tested materials was calculated from the contact angle values formed by water, formamide, ethylene glycol, and diiodomethane. Due to the incorporation of antioxidants into the PLA matrix, higher values of water contact angles were obtained than for the reference sample, which also proves their lower wettability, but on the other hand, greater resistance to oxidation processes. In the following study, a similar tendency was noticed for PLA and PLA-CBD samples before weathering.

Based on the surface energy measurements, changes due to weathering aging were monitored for all samples by increasing the values of the SFE, which signifies a degradation of the polymer matrix. Moreover, it is likely that CBD was labile and migrates to the polymer surface and increases the surface energy value through the presence of the polar groups. Comparing two polymers that were applied in this research, ethylene–norbornene copolymer exhibited better resistance to degradation processes than polylactide, and in both cases, the addition of cannabidiol extract proved to be successful as a natural stabilizer, which preserved the surface properties of the tested materials. However, Rudawska et al. (2009) [43] described the analysis for determining surface free energy uncertainty by the Owen–Wendt method, and they stressed that there are many factors that have an influence on the accuracy of measured contact angle values, e.g., size of the drop, temperature, surface rigidity, surface roughness, homogeneity of surface (and surface layer), impurities, humidity, and type of measured liquid. Therefore, to assess the antioxidant effect of CBD extract, other tests were also performed.

**Figure 2:**
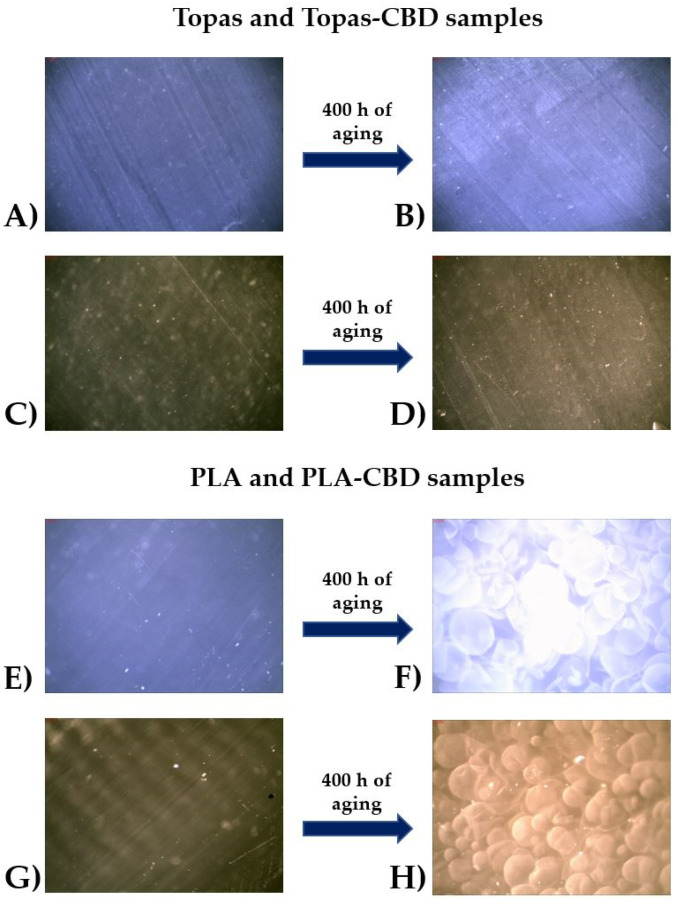
Morphology analysis with an optical microscope at 130× magnification of (**A**) ethylene-norbornene copolymer (Topas) before aging, (**B**) Topas after 400 h of weathering, (**C**) Topas-CBD reference sample, (**D**) Topas-CBD after 400 h of weathering, (**E**) polylactide (PLA) before aging, (**F**) PLA after 400 h of weathering, (**G**) PLA-CBD reference sample, (**H**) PLA-CBD after 400 h of weathering, respectively.

Furthermore, to give a clearer view of the changes in surface properties of Topas and PLA samples, the surface morphology was observed by using an optical microscope at 130× magnification, and pictures that were taken before and after 400 h of aging are presented in Figure 2. Increased temperature, radiation, and humidity, i.e., factors to which the samples were exposed during weather-aging did not cause significant changes in the surface properties of the Topas samples, but in the case of PLA, the extensive degradation of the polymer matrix was observed that probably led to the chain scission process of the material and formed oligomers and monomers. Moreover, as can be seen in Figure 2, the degradation of polylactide resulted in the formation of new rough surfaces. These samples were also characterized by considerable brittleness, which made them unsuitable for further use. The same tendency was noticed by Mysiukiewicz O. et al. (2019) [44] during their research related to the influence of weathering aging time of polylactide-based composites filled with linseed cake. They confirmed that elevated temperature and humidity can be a reason for the changes in the PLA structure, resulting in shrinkage and increased embrittlement. Moreover, the presence of voids and porosities can also result in cracks because of the insufficient ability to deform the PLA matrix.

As was mentioned before, according to the polylactide accelerated weathering tests, changes in its microstructure were observed. The reference sample revealed a smooth surface that is typical for the amorphous form of this polymer, whereas the material weathered for 400 h presented a typical surface morphology for semi-crystalline polymers that are deformed in a plastic way. It can be explained as the amorphous phase degradation and recrystallization of the solid-state [44]. Moreover, during the daytime cycle of weathering, the rain water was on, which means that the material was also exposed to hydrolytic degradation that leads to PLA chain cleavage of the ester bond in amorphous areas to create new compounds by the impact of water, such as alcohol and acid [45]. As was stated by Sawpan et al. (2019) [46], this process may also have an effect on increasing the crystallinity of the polymer. Additionally, after 400 h of aging, blisters appeared on the polylactide surface (Figure 2F,H) caused by the breakdown of PLA chains as a result of weathering. Another change that was noticed for polylactide samples after exposure to UV radiation, elevated temperature, and humidity was increased opacity (whitening of the surface). This phenomenon can be the reason for the higher crystallinity caused by the products formed during hydrolytic degradation and absorbed water in polylactide.

As is well known, surface morphology has a great influence on the surface properties and the contact angles of the tested materials. Spiridon et al. (2015) [47] investigated the wettability properties of PLA-based composites before and after 600 h of weather-aging. As they observed, exposure to the synergistic effects of climatic conditions has influenced the wettability of the obtained materials. The water contact angle value for the reference PLA was equal to 77.8°, whereas, after 600 h of aging, a significant decrease in this value was noticed (29.2°). The reason could be the matrix cracks and change in the surface morphology. A similar tendency can be found in this study by comparing the obtained water contact angle values for PLA samples and their surface behavior. For both pure PLA and PLA-CBD samples, the water contact angle values considerably decreased, and the polar component of the surface free energy increased, which designates that the hydrophilicity of these materials has been improved. Therefore, these results may indicate that the hydrolytic degradation was progressing, which was also confirmed by analyzing the morphology using an optical microscope.

**Figure 3:**
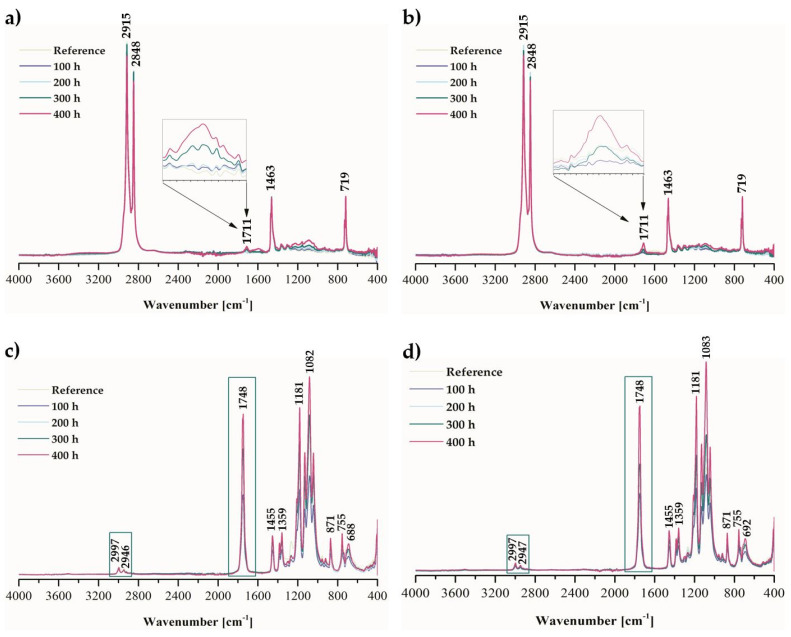
Fourier-transform infrared spectra of (**a**) pure ethylene-norbornene copolymer (Topas), (**b**) Topas with CBD extract, (**c**) pure polylactide (PLA), (**d**) PLA with CBD content, before and after 100, 200, 300, and 400 h of weather-aging.

The aging effect was also tested by using Fourier-transform infrared spectroscopy. This method identifies the functional groups in materials and structural changes that appeared due to exposure to radiation, higher temperature, and humidity during weathering. All obtained absorbance spectra within the 4000–400 cm^−1^ range for pure Topas and PLA samples and with the CBD content are presented in Figure 3. Moreover, for each material, its characteristic peaks were assigned to the specific functional groups. For ethylene–norbornene copolymer, peaks at 2915 and 2848 cm^−1^ were attributed to the CH_2_ groups, at 1711 cm^−1^ to carbonyl groups (C=O), at 1463 cm^−1^ to CH_2_ groups (bending band), and at 719 cm^−1^ to a rocking band of CH_2_. However, in the case of polylactide samples, the bands around 2997 and 2946 cm^−1^ were assigned to the stretching vibrations of -CH groups, 1748 cm^−1^ to the carbonyl carbon of the PLA ester group, 1455 and 1359 cm^−1^ to the -CH group deformation (containing symmetric and asymmetric bending), 1181, 1082, and 871 cm^−1^ to the stretching vibrations of C-O and C-C, respectively.

**Figure 4:**
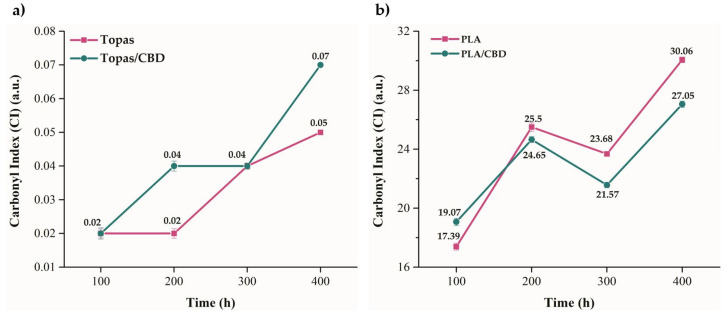
Carbonyl index (CI) values obtained (**a**) for ethylene-norbornene copolymer (Topas) and (**b**) polylactide (PLA) mixtures after weather-aging, where C=O groups were observed within the 1600–1800 cm^−1^ range and CH groups at 2915 and 2946 cm^−1^, respectively.

Furthermore, based on the Fourier-transform infrared spectroscopy (FTIR) obtained spectra, the monitoring of sample oxidation was possible by observing the changes in the peak intensity within the 1600–1800 cm^−1^ range that took place in the carbonyl band (C=O). By applying Equation (4), the carbonyl index value for each material after weathering was calculated (Figure 4). The changes due to weather-aging for Topas and Topas-CBD blends were practically imperceptible, for which the carbonyl index parameter was close to 0.1. On the other hand, the influence of elevated temperature, radiation, and humidity for PLA-based samples was more significant, and after 400 h of aging, the carbonyl index (CI) value was close to 30. Based on the carbonyl index values, FTIR analysis also confirmed the greater susceptibility of polylactide to weather-aging. Moreover, by comparing the obtained carbonyl index results for the reference sample of PLA and PLA-CBD blend after 400 h of weathering, the addition of cannabidiol slightly improved the resistance to oxidation.

**Figure 5:**
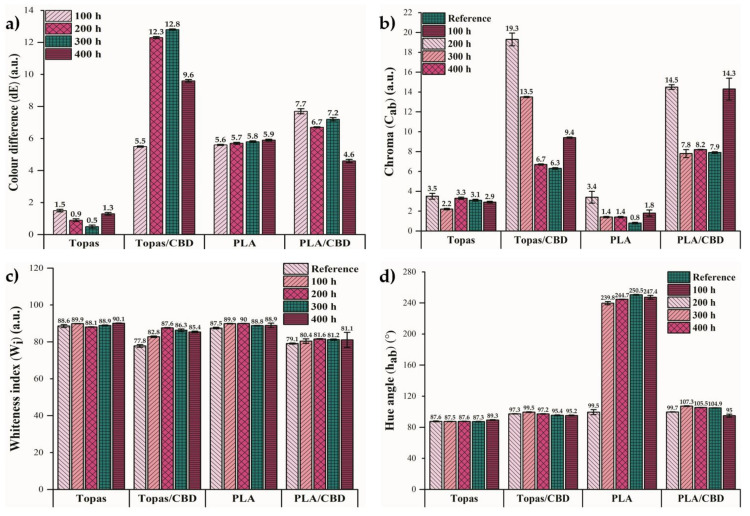
Optical properties of ethylene-norbornene copolymer (Topas) and polylactide (PLA) blends: (**a**) color difference (dE), (**b**) chroma (C_ab_), (**c**) whiteness index (W_i_), (**d**) hue angle (h_ab_) before and after 100, 200, 300, and 400 h of weathering. Presented values are the average of three measurements.

In the next part of the study, the color change in prepared samples before and after controlled weather-aging was analyzed by a UV-Vis spectrophotometer. As was mentioned before, phytochemicals are susceptible to UV radiation, which can result in their degradation process related to changes in the properties of these natural compounds. Change in color is the first visible feature of polymer degradation, but in some cases, it can be considered as an asset, e.g., when such phytochemicals can act as natural dyes or aging color indicators. Masek A. et al. (2013) [48] proposed morin hydrate as a pro-ecological antioxidant that can be applied in polyolefin polymers, and in the authors’ opinion, this compound not only protects the material against aging but also fulfills the role of a natural dye. Singh S. et al. (2018) [49] presented the possibility of using anthocyanins as natural pigments from plant sources that have a great potential as color indicators in smart packaging systems. The color of anthocyanins is strongly dependent on their structure, temperature, pH, UV irradiation, and presence of oxidative factor. As a result of this color instability, they can act as the color indicators in packaging applications and are particularly usable to control the food quality. In the following paper, the influence of weather-aging on the color change in Topas and PLA samples with cannabidiol content was examined. For each sample, three measurements were performed. Figure 5 shows the obtained results of (a) color change, (b) chroma values, (c) whiteness index, and (d) hue angle values for all samples before and after aging.

During weathering, the samples were simultaneously exposed to increased temperature, humidity, and UV radiation. Therefore, the color change in the tested materials comes from a combination of three factors, but among them, UV radiation has the greatest influence. By comparing the obtained results of color difference (dE), much higher values were found for the samples containing the CBD extract. After 200 and 300 h of controlled aging, the color change for Topas-CBD samples was close to 13, whereas for PLA-CBD blends was close to 7. By analyzing the chroma values for these mixtures, the inverse tendency can be noticed by a significant decrease in their degree of saturation. Moreover, for the samples with CBD addition, no significant changes in the whiteness index and hue angle values were observed. On the other hand, for neat PLA samples after 100, 200, 300, and 400 h of weathering, a significant increase in the hue angle parameter was observed (~245°), for which the values of a* and b* coordinates obtained in the CIE-Lab space were negative. The positive value of a* indicates a red color, whereas negative means green. However, the positive value of b* coordinate signifies yellow and negative, blue. Therefore, weathering has the influence of shifting the color of pure PLA towards red, as indicated by the decrease in the value of a* parameter by 0.17, and towards blue, as indicated by the decrease in b* parameter by 5.01 after 400 h of aging. Moreover, the change in the hue angle value of pure PLA was greater because it was not protected in any way. Although the CBD extract did not significantly affect the antioxidation of composites during the analysis of morphology, energy changes, or FTIR, its molecules may make it stable in itself, which allows this optical property to be retained. The reason for the color change observed mainly for samples with cannabidiol extract can be the lower resistance of plant origin compounds to UV radiation. In the available literature, it has been highlighted that phytochemicals are able to change coloration when they are exposed to UV radiation, which is a general appearance in the natural environment [50]. Furthermore, changes in the color of polymeric materials can be due to the oxidation process that alters the molecular structure of antioxidants. In this analysis, the CBD extract proved to be effective as a potential aging color indicator that informs about changes during the service life of a polymer product.

### 2.2. Thermal Analysis of Ethylene-norbornene Copolymer (Topas) and Polylactide (PLA) Composites with Cannabidiol (CBD) Extract

**Figure 6:**
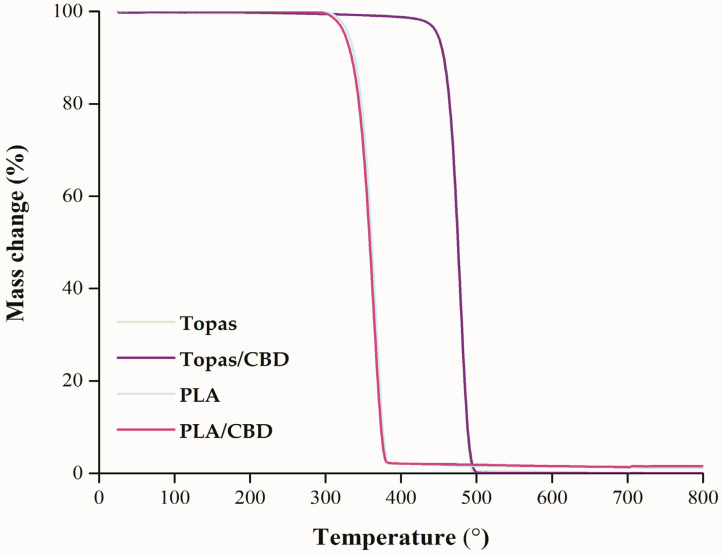
Thermogravimetric analysis (TGA) analysis of ethylene-norbornene copolymer (Topas) and polylactide (PLA) samples before aging that were heated from 25 to 800 °C.

To assess the thermal stability of Topas and PLA blends with the cannabidiol content, a thermogravimetric analysis (TGA) was performed (Figure 6). The obtained results of T_2%_, T_5%_, T_10%_, T_20%_, T_50%_, T_70%_, and T_90%_ for all samples before weather-aging are presented in Table 2 which corresponded to the mass loss of 2%, 5%, 10%, 20%, 50%, 70%, and 90%, respectively.

For both polymers, the one-step thermal decomposition can be observed in Figure 6. Ethylene–norbornene copolymer was characterized by an initial temperature that was around 432 °C and the final degradation temperature that was close to 490 °C, whereas for polylactide, the obtained value of initial temperature was around 320 °C, and the maximum temperature was close to 375 °C. Additionally, the results obtained in Table 2 showed that the addition of cannabidiol extract to both polymer matrices did not improve their thermal stability.

On the other hand, the addition of cannabidiol to Topas and PLA blends resulted in a significant increase in their oxidative-induction time/temperature parameter (OIT), at which an exothermic peak occurred in the differential scanning calorimetry (DSC) curve. The initial (onset) time of the Topas samples oxidation is presented in Table 3, whereas the onset and endset temperatures of the PLA oxidation peaks are shown in Table 4. An extension of the oxidation time (t_0_) was observed for Topas containing cannabidiol extract, which was 3.40 min, while for PLA with CBD, both the initial and final oxidation temperatures increased significantly by 54.1 °C and 44.6 °C, respectively. It means that the addition of cannabidiol to polymer matrices increases their thermal stability and enhances their resistance to oxidation processes. This analysis confirmed that CBD extract can be applied as an effective natural stabilizer for polymeric materials. In the available literature, a similar tendency was noted for quercetin and silibinin used as natural stabilizers for polypropylene that significantly improved the value of OIT parameter [51].

In this study, the use of cannabidiol extract as a natural stabilizer with antioxidant properties for polymeric materials was found to be effective mainly through the analysis of the OIT parameter values. Additionally, in the case of polylactide samples, a small improvement was also observed for the carbonyl index and polar component of the SFE values. Comparing the obtained results with those available in the literature, it can be assumed that in terms of maintaining physicochemical properties, this compound works similarly to such antioxidants as hesperidin, chrysin, or naringin, which also have antioxidant properties, but on the other hand, it significantly increases the resistance of the sample to oxidation processes. Samper et al. (2013) [51] compared the oxidative-induction time/temperature values of quercetin and silibinin in the amount of 0.75 phr used in a polypropylene (PP) matrix, and their results showed that OIT of PP increased by 86.3 min and 38.4 °C as a result of quercetin addition, whereas for PP with silibinin content increased by 76.0 min and 19.4 °C, respectively. Therefore, taking into account only the OIT parameter, the effectiveness of cannabidiol extract can be compared to more active antioxidants, such as quercetin.

## 3. Materials and Methods

### 3.1. Reagents

In this research, a thermoplastic elastomer, ethylene–norbornene copolymer (TOPAS^®^ Elastomer E-140), from TOPAS Advanced Polymers^®^ (Raunheim, Germany) and polylactide (PLA) (IngeoTM Biopolymer 4043D) from Nature Works (Minnetonka, MN, USA) were used as polymer matrices. Topas is a material characterized by a melting temperature of 84 °C, whereas its Vicat softening temperature is equal to 64 °C. Moreover, it has good transparency, high purity, glass-like texture, and very low permeability to gases and water. Therefore, ethylene–norbornene copolymer is often used in cosmetics, packaging for foods, or pharmacy. However, polylactide is a biodegradable polymer, which is characterized by the following properties: Melt Flow Index (MFI) = 6 g/10 min, Tm = 145–160 °C, and Tg = 55–60 °C.As is a natural additive in the role of PLA and Topas stabilizer, cannabidiol (CBD) extract, which is one of the identified cannabinoids in cannabis plants and completely tetrahydrocannabinol (THC) free at a concentration of 1 phr (parts per hundred rubber) was used. This compound was a commercial sample applied as received.

### 3.2. Methods of Samples Preparation

To prepare the polymer blends of ethylene–norbornene copolymer, a laboratory micromixer (Brabender Lab-Station) from Plasti-Corder (Duisburg, Germany) equipped with a Julabo cooling system was used. The processing temperature was equal to 110 °C and the mixing time was 30 min with the rotation speed of 60 rpm/min. Then, the samples were vulcanized in the hydraulic press for 10 min at a temperature of 160 °C and under pressure equal to 125 bar. To prevent a contact between the polymeric samples and steel molds, special Teflon sheets were applied.

In the case of polylactide (PLA) granules that were dried at a temperature of 50 °C for 12 h, an extrusion process was performed by using a laboratory extruder from Zamak Maercator (Skawina, Poland) that works in the horizontal position and is equipped with a screw characterized by a diameter of 25 mm. First, the PLA granules were mixed with CBD extract in a beaker and then poured into the extruder. The processing temperature was equal to 180 °C with the rotation speed of 40 rpm and under the pressure of 17 atm. The final shape of samples was similar to long flat stripes that were molded by a cylinder head and cooled at room temperature. Table 5 presents the compositions of all prepared mixtures.

### 3.3. Weather-Aging

To assess the aging behavior of ethylene-norbornene copolymer (Topas) and polylactide (PLA) blends with cannabidiol (CBD) extract, weathering tests were performed by using a Weather-Ometer Ci 4000 chamber with a xenon lamp from Atlas Material Testing Technology LLC (Chicago, IL, USA) in accord with the PN-EN 4892-2 standard Plastics-Methods of exposure to laboratory light sources-Part 2: Xenon arc lamps. Weather-aging consisted of two recurrent segments, daytime and nighttime, respectively, where each spanned 900 min. During the day cycle, the samples were subjected to the following conditions: radiation intensity equal to 60.4 W/m^2^ over a λ range of 300 to 400 nm, humidity 80% with the rain water on, and temperature of 60 °C, while for the nighttime cycle, the radiation was disabled, the temperature was equal to 50 °C, and humidity was 70% with the rain water off. Special stainless-steel frames were used, in which samples were placed in the chamber and aged for 100, 200, 300, and 400 h, respectively.

### 3.4. Measurement Methods

#### 3.4.1. Surface Free Energy (SFE)

The surface free energy of ethylene-norbornene copolymer and polylactide mixtures with cannabidiol extract was measured by using a goniometer OCA 15EC from DataPhysics Instruments GmbH (Filderstadt, Germany) and software module SCA 20. This research was based on the Owens–Wendt–Rabel–Kealble (OWRK) method and consisted of the contact angles determination for tested samples by applying polar and non-polar fluids (distilled water, diiodomethane, and ethylene glycol). For each material before and after weather-aging, 10 contact angles of the three fluids were marked. SFE parameter of the solids (γ_s_) is the sum of polar and dispersive constituents, Equation (1) [52]:

(1) $$\gamma_{s}=\gamma_{s}^{P}+\gamma_{s}^{D}$$

where γ_s_ is the surface free energy (SFE) [mJ/m^2^], $\gamma_{s}^{P}$ is the polar component of the SFE [mJ/m^2^], and $\gamma_{s}^{D}$ is the dispersive component of the SFE for the tested samples (mJ/m^2^).

To calculate the polar and dispersive constituents following the OWRK method, Equations (2) and (3) were applied [53]:

(2) $${\left(\gamma_{s}^{P}\right)}^{0,5}=\frac{\gamma_{w}\cdot \left({\cos\theta }_{w}+1\right)-2\cdot \sqrt{\gamma_{w}^{D}\cdot \gamma_{s}^{D}}}{2\cdot \sqrt{\gamma_{w}^{P}}}$$

(3) $${\left(\gamma_{s}^{D}\right)}^{0,5}=\frac{\gamma_{D}\cdot \left({\cos\theta }_{D}+1\right)-\sqrt{\frac{\gamma_{D}^{P}}{\gamma_{w}^{P}}\cdot \gamma_{w}\cdot \left({\cos\theta }_{w}+1\right)}}{2\cdot \left[\sqrt{\gamma_{D}^{D}-\sqrt{\gamma_{D}^{P}\cdot \frac{\gamma_{w}^{D}}{\gamma_{w}^{P}}}}\right]}$$

where $\gamma_{w}$ is the surface free energy for water that was equal to 72.6 (mJ/m^2^), $\gamma_{D}$ is the surface free energy for diiodomethane that was equal to 50.8 (mJ/m^2^), $\gamma_{w}^{D}$ and $\gamma_{w}^{P}$ are the dispersive (21.6 mJ/m^2^) and polar (51.0 mJ/m^2^) components for water, respectively,$\;\gamma_{D}^{D}$ and $\gamma_{D}^{P}$ are the dispersive (48.5 mJ/m^2^) and polar (2.3 mJ/m^2^) constituents for diiodomethane, respectively.

#### 3.4.2. Surface Morphology Analysis

An assessment of the ethylene-norbornene copolymer (Topas) and polylactide (PLA) microstructure before and after 400 h of weather-aging was possible using an optical, stereoscopic microscope MZ 6 from Leica Microsystems (Germany) and the Opta View program (Poland) for image analysis. In the case of pure Topas and PLA, a white light to illuminate the samples was applied, whereas, for Topas and PLA samples with cannabidiol (CBD) content, a yellow light was used. All of the analyses were performed at room temperature, and the magnification applied during this test was about 130×.

#### 3.4.3. Fourier-Transform Infrared Spectroscopy

To assess the structural changes that appeared due to the aging, the Fourier-transform infrared spectroscopy (FTIR) method was performed for all samples before and after weathering. The FTIR absorbance spectra were obtained by using a Nicolet 6700 FTIR spectrophotometer (Thermo Scientific) equipped with Smart Orbit ATR sampling accessory and the measurement range was between 4000 and 400 cm^−1^ with 64 scans at 4 cm^−1^ resolution. Furthermore, this analysis enabled the monitoring of material oxidation by observing changes that take place in the carbonyl (C=O) band. Therefore, based on the FTIR spectrum, a carbonyl index (CI) value was calculated for each sample before and after weather-aging with Equation (4) [54]:

(4) $$\mathrm{Carbonyl}\;\mathrm{Index}=\frac{I_{C=O}}{I_{C–H}}$$

where I_(C=O)_ is the peak intensity that corresponds to the C=O groups (~1740 cm^−1^), and I_(C–H)_ is the peak intensity that represents the -CH groups (~1920 cm^−1^).

#### 3.4.4. Change in Color Measurements

Color difference analysis for the ethylene-norbornene copolymer (Topas) and polylactide (PLA) blends with cannabidiol (CBD) extract was realized by applying the UV-VIS CM-3600d spectrometer from Konica Minolta Sensing, Inc. (Osaka, Japan) in accordance with the PN-EN ISO 105-J01 standard. The spectral range of the measurement was 360 to 740 nm, and the radiation source was made up of four xenon tube pulses. For each material, three measurements were performed. Furthermore, the CIE-Lab scale was used to interpret the obtained results. In general, this system allows describing the color of the tested materials by three coordinates: a* and b* axes, for which a positive value of a* means a red color, whereas negative means green. In the case of the b* coordinate, the positive value represents a yellow color, and negative is blue. The third parameter is the L axis, which indicates a lightness index with the maximum value of 100 that corresponds to black. Then, based on the obtained results, the color difference (dE), whiteness index (W_i_), chroma (C_ab_), and hue angle (h_ab_) values were determined with the following Equations (5)–(8) [55]:

(5) $$\mathrm{dE}=\sqrt{{\left(\Delta a\right)}^{2}+{\left(\Delta b\right)}^{2}+{\left(\Delta L\right)}^{2}}$$

(6) $$W_{i}=100-\sqrt{a^{2}+b^{2}+{\left(100-L\right)}^{2}}$$

(7) $$C_{\mathrm{ab}}=\sqrt{a^{2}+b^{2}}$$

(8) $$h_{\mathrm{ab}}=\{\begin{array}{c} \mathrm{arctg}\left(\frac{b}{a}\right)\;\mathrm{as}\;a>0\wedge b>0\\ 180{}^{\circ}+\mathrm{arctg}\left(\frac{b}{a}\right)\;\mathrm{as}\;\left(a<0\wedge b>0\right)\vee (a<0\wedge b<0\\ 360{}^{\circ}+\mathrm{arctg}\left(\frac{b}{a}\right)\;\mathrm{as}\;a>0\wedge b<0 \end{array}$$

#### 3.4.5. Thermogravimetric Analysis (TGA)

Thermogravimetry is a thermal analysis that enables monitoring the mass change of the sample against temperature or time in a controlled environmental furnace and assessing the fastest or delayed thermal degradation process [56]. In this study, the thermal stability of the tested materials was determined by using a Mettler Toledo^®^ apparatus (Greifensee, Switzerland). The mass sample of 7–9 mg was measured on a microbalance, and then the samples were heated from 25 to 800 °C under an argon atmosphere with a heating rate of 10 °C/min.

#### 3.4.6. Oxidative-Induction Time (OIT)

The oxidative-induction time (OIT) test was performed by applying a Mettler Toledo Differential Scanning Calorimeter apparatus (Greifensee, Switzerland) according to the PN EN 728:1999 standard, “Plastic piping system and ducting system. Polyolefin pipes and fitting determination of induction time” and the ISO 11357-6:2002 standard “Plastics-DSC-Part 6: Determination of oxygen induction time”. This test allows marking a degree of thermal stabilization. Prepared samples with a weight of 6–8 mg were placed in an aluminum crucible with a volume of 100 μL and subjected to heating from the room to the investigation temperature. The OIT value was determined as the time between the melting of the material and the beginning of its decomposition. Ethylene-norbornene copolymer (Topas) and Topas-CBD blend were heated to the research temperature equal to 210 °C under an air atmosphere. The measurement time was 50 min. On the other hand, for polylactide (PLA) samples, this test consisted of four different segments:

- Heating from 0 to 200 °C for 15 min under an argon atmosphere at a flow rate of 50 mL/min;
- Heating in the investigation temperature, 200 °C for 10 min under an argon atmosphere at a flow rate of 50 mL/min;
- Cooling from 200 to 0 °C for 20 min under an argon atmosphere at a flow rate of 50 mL/min;
- Heating from 0 to 350 °C for 30 min under an air atmosphere at a flow rate of 50 mL/min.

## 4. Conclusions and Future Research Directions

In this study, the influence of cannabidiol (CBD) extract from cannabis plants was examined as a potential natural stabilizer to polymers. The obtained results showed that it can be successfully used as an antioxidant because for ethylene–norbornene copolymer and polylactide samples with CBD content, an increase in the oxidative-induction time/temperature (OIT) parameter value was observed, which could be caused by the content of two hydroxyl groups in its structure. For Topas containing cannabidiol, the oxidation induction time was longer by 3.40 min in comparison to the neat sample, whereas for polylactide (PLA), both the initial and final oxidation temperatures increased significantly by 54.1 °C and 44.6 °C, respectively. Furthermore, after 200 and 300 h of controlled aging, the color change for Topas-CBD samples was close to 13, whereas for PLA-CBD mixtures, it was close to 7. Therefore, this additive can also be considered as an aging color indicator, which allows controlling the changes that occur during the lifetime of the polymeric products. Based on the obtained results in the study, the subsequent research will focus on the effect of the used amount of cannabidiol extract on polymer antioxidant properties. Moreover, the cytotoxicity tests of the products obtained during the weather-aging would be an interesting approach to assess their safety for humans and the environment.

## Figures and Tables

**Table 1:** Contact angle values determined by using distilled water, ethylene glycol, and 1,4-diiodomethane for ethylene–norbornene copolymer (Topas) and polylactide (PLA) mixtures before and after weather-aging. Each value is the average of 10 determined contact angles.

**Liquid**	Contact Angle after Weathering Aging (°)				
	Reference	100 h	200 h	300 h	400 h
Topas					
Water	87.6 ± 0.79	90.6 ± 6.61	98.2 ± 1.01	97.8 ± 1.73	97.6 ± 0.97
Diiodomethane	61.6 ± 1.43	59.9 ± 0.74	55.4 ± 1.89	62.5 ± 1.73	53.8 ± 1.68
Ethylene glycol	63.3 ± 1.72	65.4 ± 1.79	74.9 ± 1.04	75.0 ± 0.66	69.5 ± 2.05
Topas/CBD					
Water	71.2 ± 0.92	97.3 ± 1.08	85.9 ± 1.08	92.9 ± 1.04	97.1 ± 0.99
Diiodomethane	55.4 ± 0.95	51.1 ± 0.74	47.0 ± 0.56	48.6 ± 0.96	49.5 ± 1.28
Ethylene glycol	48.1 ± 2.29	71.9 ± 1.88	58.0 ± 1.32	65.9 ± 1.17	69.4 ± 1.09
PLA					
Water	82.1 ± 0.84	86.1 ± 0.54	76.0 ± 0.95	75.0 ± 1.25	65.1 ± 0.73
Diiodomethane	54.2 ± 1.17	51.6 ± 1.74	44.0 ± 1.32	46.9 ± 2.50	41.3 ± 3.50
Ethylene glycol	57.8 ± 1.47	61.2 ± 1.45	49.5 ± 0.85	48.0 ± 1.10	42.7 ± 1.65
PLA/CBD					
Water	87.8 ± 1.44	78.9 ± 1.62	76.8 ± 1.76	71.6 ± 1.01	67.9 ± 2.04
Diiodomethane	47.6 ± 2.23	46.8 ± 1.46	51.4 ± 2.68	40.3 ± 1.23	33.8 ± 2.43
Ethylene glycol	58.9 ± 1.34	51.1 ± 1.28	48.2 ± 0.84	50.3 ± 1.84	40.7 ± 3.32

**Table 2:** Temperatures of the mass loss of tested materials, where T_x%_ is a temperature at which the mass change is x% (2, 5, 10, 20, 50, 70, 90%, respectively).

Mixture	Temperatures of Mass Change (°C)						
	T_2%_	T_5%_	T_10%_	T_20%_	T_50%_	T_70%_	T_90%_
Topas	432.5	449.2	456.7	464.2	475.8	480.8	487.5
Topas/CBD	429.2	448.3	456.7	464.2	475.0	480.8	487.5
PLA	320.0	330.0	338.3	347.5	360.8	367.5	375.0
PLA/CBD	314.2	325.0	334.2	344.2	358.3	365.0	373.3

**Table 3:** Oxidative-induction time (OIT) values of ethylene-norbornene copolymer (Topas)-based samples before aging and their energy of oxidation.

Mixture	Oxidative-Induction Time (min)	Energy of Oxidation (J/g)
Topas	5.09	170
Topas/CBD	8.49	223

**Table 4:** Oxidative-induction temperature values of polylactide (PLA) and PLA with cannabidiol (CBD) content before weathering and their oxidation energy.

Mixture	Oxidative-Induction Temperatures (°C)		Energy of Oxidation (J/g)
	Onset (°C)	Endset (°C)	
PLA	222.0	261.3	11
PLA/CBD	276.1	305.9	8

**Table 5:** The composition of ethylene-norbornene copolymer (Topas) and polylactide (PLA) based mixtures with the natural additive, cannabidiol extract.

Mixture	Weight Composition (phr)		
	Topas	PLA	CBD Extract
1	100	-	-
2	100	-	1
3	-	100	-
4	-	100	1

## Data Availability

Not applicable.

## Abbreviations

CBD	Cannabidiol extract obtained from Cannabis Plants
PLA	Polylactide
COCs	Cyclic Olefin Copolymers
Topas	Ethylene–Norbornene Copolymer
PS	Polystyrene
PP	Polypropylene
PE	Polyethylene
PHAs	Polyhydroxyalkanoates from aliphatic polyesters group
BHA	Butylated Hydroxyanisole
BHT	Butylated Hydroxytoluene
THC	Tetrahydrocannabinol
FTIR	Fourier-transform Infrared Spectroscopy
OIT	Oxidation Induction Time/Temperature
SFE	Surface Free Energy [mJ/m^2^]
CI	Carbonyl Index [–]
TGA	Thermogravimetric Analysis
DSC	Differential Scanning Calorimetry
